# Effect of downregulation of serum MMP-3 levels by traditional Chinese medicine ingredients combined with methotrexate on the progression of bone injury in patients with rheumatoid arthritis: A protocol for a systematic review and meta-analysis

**DOI:** 10.1097/MD.0000000000022841

**Published:** 2020-10-23

**Authors:** Yue Sun, Yucheng Huang, Tiantian Chen, Xueping Li, Jiayi Chen, Zhuozhi Wang, Kexin Lin, Yongxiang Gao, Lisha He

**Affiliations:** a College of Basic Medicine; b College of Clinical Medicine; c Department of Rheumatology, Hospital of Chengdu University of Traditional Chinese Medicine; d College of Medical and Life Sciences, Chengdu University of Traditional Chinese Medicine, Chengdu, P.R. China.

**Keywords:** bone injury, MMP-3, MTX, RA, traditional Chinese medicine ingredients

## Abstract

**Background:**

A large number of clinical studies have confirmed that after treatment with traditional Chinese medicine components such as sinomenine (SIN), the matrix -metalloproteinase3 (MMP-3) level of patients with rheumatoid arthritis (RA) shows a significant decrease, whereas MMP-3 can be involved in degrading bone matrix in humans, so in the progression of bone and joint injury in patients with RA, serum MMP-3 can be used as an important biochemical marker. The traditional Chinese medicine components commonly used in clinical practice include total glucosides of paeony (TGP), SIN, and tripterygium glycosides, which have the characteristics of disease-modifyinganti-rheumatic drugs and non-steroidal anti-inflammatory drugs, while they can reduce the toxic side effects of methotrexate (MTX), and their combination with other drugs such as MTX and leflunomide (HWA486) has become an important regimen for the treatment of RA in clinical practice. Therefore, we designed this study protocol to evaluate the adjuvant effect of commonly used traditional Chinese medicine components combined with MTX in the treatment of osteoarticular injury in RA.

**Methods:**

The search time was set from January 2000 to September 2020 in this study. EMBASE database, Cochrane Library, PubMed, Web of Science, Science Direct, Chinese National Knowledge Infrastructure, China Biology Medicine disc (CBM), Chinese Scientifific Journals Database (VIP), and Wanfang Database were used as search sources to select the traditional Chinese medicine components that reduce MMP-3 and use MTX in the treatment of RA. Clinical randomized controlled trials were used, and inclusion criteria and exclusion criteria were set for screening. In this study, MMP-3, erythrocyte sedimentation rate (ESR), C-reactive protein (CRP), cyclic peptide containing citrulline (CCP) and rheumatoid factor (RF) were used as the main outcomes, and the improvement of Disease Activity Score 28 (DAS28), joint bone mineral density, Clinical Disease Activity Index (CDAI), and other clinically relevant symptoms was selected as the secondary outcomes. Revman software version 5.3 was used for statistical analysis of data and risk assessment of deviation in this meta-analysis. In this study, one researcher performed study direction selection, literature inquiry, and literature download, and 2 independent reviewers performed literature data extraction and literature quality assessment. Dichotomized data are expressed as relative risk, continuous data are expressed as mean difference or standard mean difference, and finally fixed-effect model or random-effect model is used for synthesis according to the heterogeneity of data.

**Results:**

To evaluate the effect of downregulation of MMP-3 level by traditional Chinese medicine components combined with MTX on the progression of bone injury in patients with RA by serum MMP-3, ESR, CRP, CCP, and RF.

**Conclusion:**

This study protocol can be used to evaluate the efficacy and safety of traditional Chinese medicine components combined with MTX in the treatment of bone injury in patients with RA.

**Ethics and dissemination:**

This study is a secondary study based on the published clinical research; therefore, approval from an ethics committee is not required for this study. In accordance with the Preferred Reporting Items for Systematic Reviews and Meta-Analysis Protocol (PRISMA-P), the results of this study will be published in peer-reviewed scientific journals and conference papers.

**Registration number:**

is INPLASY202090064.

## 1 Introduction

Existing studies have shown that higher levels of matrix metalloproteinase3 (MMP-3) and C-reactive protein (CRP) may be important risk factors for atlantoaxial subluxation or semiluxation in rheumatoid arthritis (RA) patients.^[1]^ Some researchers have observed that asymptomatic cervical instability and other manifestations of cervical spine involvement often occur in patients with RA.^[2]^ Patients with RA are at more than twice the risk of vertebral fracture and nonvertebral fractures than healthy individuals,^[3]^ whereas in patients with advanced RA, the effect of reduced bone strength shows a significant correlation with the occurrence of osteoporotic fractures. MMP-3 is a tissue degrading enzyme, and human serum MMP-3 levels are used as an outcome measure of disease activity in patients with RA and are one of the commonly used biomarkers in rheumatology and immunology.^[3,4]^ It is now widely accepted that activation of the RANK/RANKL/OPG system causes bone destruction in patients, and MMP-3 is a receptor activator of this system.^[5]^ In addition, the increase in MMP-3 was also associated with signal transducer and activator of transcription-1 (STAT1) and STAT3 synovial phosphorylation.^[6]^ The study data showed that the serum MMP-3 level in RA patients was positively correlated with the increase of joint swelling count, and also significantly correlated with erythrocyte -sedimentation rate (ESR), CRP, and Disease Activity Score 28 (DAS28).^[7]^ Therefore, we believe that the rise in serum MMP-3 levels would increase the possibility of bone damage in patients with RA.

In addition, studies have shown that the diagnostic specificity of MMP-3 for patients with early-RA is not high enough, but serum MMP-3 can still be used as an effective indicator to evaluate the difference in the prognosis of joint destruction in patients with RA, reflecting the disease activity and treatment response of patients with moderate to severe RA.^[8]^ Methotrexate (MTX) is commonly used to treat RA in clinical practice. What for patients who do not respond well to MTX? Studies have shown that RA can be treated by triple therapy (MTX + anisulfapyridine + hydroxychloroquine) or MTX plus infliximab therapy.^[9]^ Nowadays, there may be more options in clinical practice because western medicine simple therapy is not completely risk-free, for example, the choice of traditional Chinese medicine preparations combined with MTX to inhibit MMP-3 levels may be an effective regimen with a protective effect on the patient's bone and joint. In clinical trials of sinomenine (SIN) combined with MTX in the treatment of RA, there is evidence that the corresponding disease activity indicators are reduced while MMP-3 is reduced, and the corresponding imaging studies are also improved,^[10]^ which may mean that we can intervene in the process of bone injury in RA patients by regulating MMP-3 levels. Therefore, in the face of a large body of clinical evidence, we hypothesize that traditional Chinese medicine ingredients combined with MTX play a beneficial protective role in the progression of bone injury in patients with RA. However, previous evidence-based medicine failed to confirm whether the intervention effect of traditional Chinese medicine components combined with MTX on the progression of bone injury in patients with RA was significant.

Therefore, this study was designed based on the auxiliary role of traditional Chinese medicine components combined with MTX in the progression of bone injury in patients with RA fully assessed by serum MMP-3 and RA activity and other indicators. The effectiveness and safety of traditional Chinese medicine components for the treatment of RA are confirmed, providing a higher quality evidence-based medicine basis for patients with RA.

## 2 Methods

### 2.1 Registration

The system review scheme has been registered on the INPLASY website (https://inplasy.com/inplasy-2020-9-0064/) and the registration number is INPLASY202090064. In this study, the protocol will be implemented in accordance with the methodology described in the Cochrane Manual for systematic Evaluation of interventions^[11]^ and reported in accordance with the PRISMA-P.^[12]^ If we make any adjustments during this study, we will contact with the INPLASY Administrator to update this database.

### 2.2 Inclusion criteria

#### 2.2.1 Types of study

All relevant randomized controlled trials (RCTs) published in English and Chinese on traditional Chinese medicine components combined with MTX in the treatment of RA could be included, do not distinguish whether they are single-blind, double-blind, or non-blind. Non-randomized controlled trials (RCTs), reviews, animal experiment, case reports, expert experience, and duplicate publications will be excluded.

#### 2.2.2 Participants

The patients with RA of different ages can be included in the study, regardless of nationality, sex, race, occupation and education. However, bone injury caused by other causes, such as degenerative arthritis, suppurative arthritis, gouty arthritis, ankylosing spondylitis, reactive arthritis, osteoarthritis, and synovitis was excluded.

#### 2.2.3 Intervention

The experimental group was treated with traditional Chinese medicine components combined with MTX, and the control group was treated with MTX. Neither the treatment group nor the control group received additional anti-RA and bone-protection treatment.

#### 2.2.4 Outcomes

In this study, the main primary outcomes were the level of serum MMP-3 and the improvement of ESR, CRP, CCP, RF in patients with RA, and the ACR20 score was calculated according to the above indexes.

Additional outcomes such as the improvement of DAS28, bone mineral density (BMD), CDAI, and other clinical related symptoms were used as secondary outcomes.

### 2.3 Search strategy

This meta-analysis was conducted from January 1, 2000 to September 2020, manually searching the Cochrane Library, PubMed, EMBASE, Science Direct, GOV (ClinicalTrials.gov/), European Drug Administration (EMA) (www.ema.europa.eu/ema/), World Health Organization (WHO), International Clinical trial Registration platform (www.wh.int/ICTRP), Web of Science, Chinese National Knowledge Infrastructure, China Biology Medicine disc, VIP, and Wanfang database. In addition, we searched the corresponding websites for clinical trial registration and gray literature on traditional Chinese medicine ingredients combined with methotrexate in the treatment of RA. We use a search strategy of a combination of a subject term and free term, which is decided by all commentators. The key words include: MMP-3, sinomenine (SIN), tripterygium wilfordii polyglycosides (GTW), MTX, bone injury, inflammatory factors, total glucosides of paeony (TGP), RA, bone destruction, synovial injury, ESR, CRP, CCP, RF.

### 2.4 Data collection and analysis

#### 2.4.1 Studies selection

**Figure 1:**
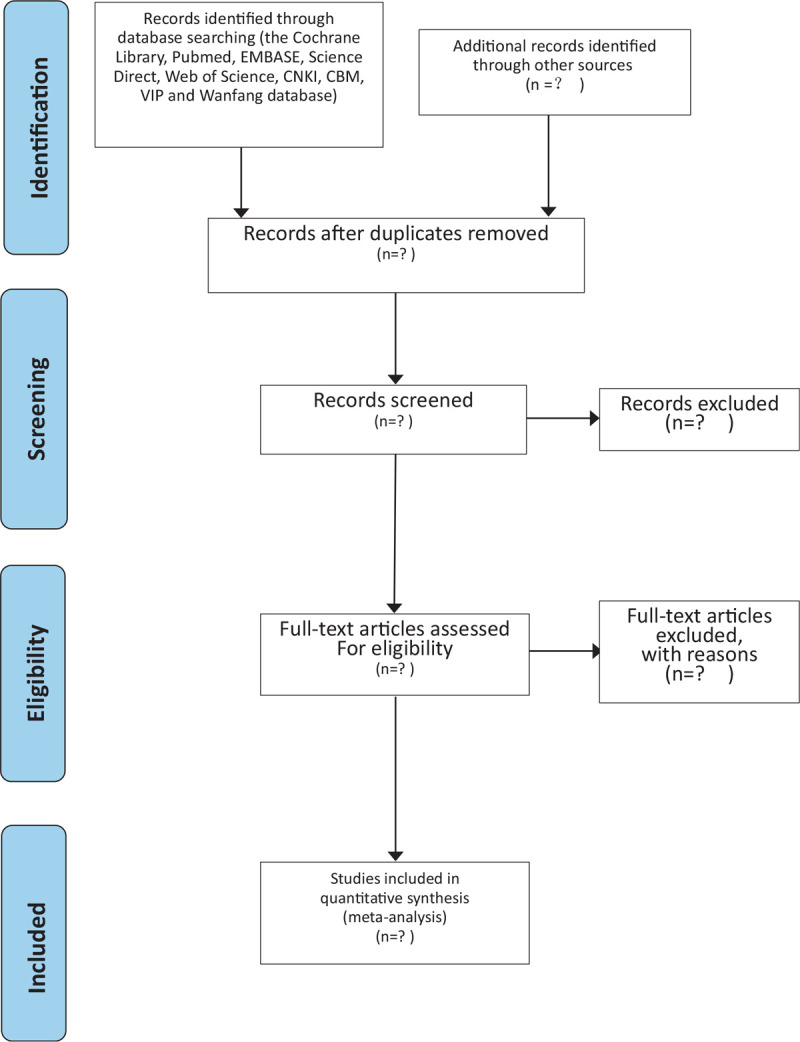
Flow diagram of the study selection process.

We put all the documents into the document management software-EndnoteX9, and the 2 reviewers (HYC, LKX) independently consult the titles and abstracts of the literature according to the pre-established inclusion and screening criteria, and preliminarily eliminate the references that do not meet the requirements. Finally, 2 independent reviewers read the specific contents of each reference carefully to further determine whether the remaining references meet the requirements of data analysis. Differences are resolved by consensus, and if dissenting opinions arise in the above process, they will be reviewed by a third-party assessor (SY). The whole selection process will be represented by a PRISMA flow diagram (Fig. 1).

#### 2.4.2 Data extraction and management

According to the required indicators, we have made EXCEL tables that meet the requirements before data extraction, which can be utilized for the input and analyze data. Two independent reviewers (HYC, LKX) can include primary and secondary outcome indicators that meet the requirements by filling in the data, and if differences arise, agreement can be reached through discussion with third-party reviewers (SY). The main data extracted by EXCEL table are as follows: title, author, year, place of attribution, age, sex, course of the disease, sample size, intervention, primary/secondary outcome indicators, follow-up, adverse reactions, and so on. If we find anything controversial, we can contact the correspondent author directly for more detailed information. The above information will eventually be cross-checked by the 2 reviewers.

#### 2.4.3 Assessment of risk of bias in included studies

We use the deviation risk (ROB) assessment tool provided by the Cochrane manual to assess the offset risk of the included RCTs quality. The evaluation contents include: blindness of participants and personnel, blindness of result evaluation, generation of random sequence, concealment of distribution, incomplete result data, selective result report, and other sources of bias. We will rate the quality of the above content, using 3 levels: “low bias,” “high bias,” and “unclear bias.” Any differences between the reviewers of both parties will be analyzed and agreed upon by the third-party reviewer (SY).

#### 2.4.4 Strategy for data synthesis

##### 2.4.4.1 Data analysis

The Review Manager5.3 provided by the Cochrane Collaboration will be used for data synthesis and meta-analysis. For bivariate data, we use the effect scale index and relative risk ratio of 95% confidence interval (95% CI), whereas the continuous data are represented by mean difference or standardized mean difference and 95% CI. The 95% CI depends on whether the measurement scale is consistent or not. When *P* < 0.01, the data are considered to be statistically significant.

##### 2.4.4.2 Assessment of heterogeneity

A *χ*^2^ test and *I*^2^ test are used to determine whether there is heterogeneity. If *I*^2^ <50% *P* > 0.1, we can think that there is no heterogeneity in the data analysis, then choose the fixed effect model comprehensive data. If *I*^2^ >50% *P* < 0.1, indicating that there is statistical heterogeneity, the random-effect model is used for analysis. Finally, the subgroup analysis was carried out according to the different causes of heterogeneity. If meta-analysis cannot be performed, a general descriptive analysis can be taken.

#### 2.4.5 Subgroup analysis

If the results are heterogeneous, we will take a subgroup analysis of possible factors that may lead to heterogeneity, such as the sex, age, race, drug dose, and MMP-3 levels.

#### 2.4.6 Sensitivity analysis

Sensitivity analysis can be carried out when the subgroup analysis is not satisfactory, and it is mainly used to evaluate the robustness of the main results. Its main operation is to eliminate the low-level quality research one by one, and then use meta-analysis software to merge new data, and finally compare with the previous results to judge the difference in sensitivity.

#### 2.4.7 Grading the quality of evidence

In the review of this study, the strength of the research evidence will be assessed by the “Grades of Recommendations Assessment, Development and Evaluation (GRADE)” standard.^[13]^ To achieve transparency and simplify the quality of the evidence will be divided into 4 levels: high, medium, low, and very low.

## 3 Discussion

RA is an autoimmune disease, which is often accompanied by cartilage destruction and joint instability.^[14,15]^ This complication is caused by local and systemic bone loss caused by increasing bone resorption in the affected joint.^[16]^ When the osteoclast pathway is activated by abnormal immune conditions, the affected joint will form periarticular osteoporosis and local bone destruction in the state of chronic inflammation.^[17]^ Some researchers used the fracture risk assessment tool (FRAX) to predict the risk of osteoporotic fracture in Chinese patients with RA for 10 years later. The results showed that the glucocorticoid use were important risk factors for osteoporotic fracture in Chinese RA patients. After adding BMD index to FRAX, the 10-year risk of osteoporotic fracture in Chinese RA patients was higher.^[18]^ This suggests that the selection of BMD markers is of significance in predicting the progression of bone injury in patients. In the treatment of RA, the use of disease-controlling of anti-rheumatic drugs and anti-osteoporotic agents combined with other biological agents to improve systemic osteoporosis is an effective treatment to control the progression of bone injury in patients with RA.^[17]^ Through these drug treatments, inflammation associated with periarticular bone destruction can be controlled, thereby reducing the risk of loose fractures in patients. TGP can improve the clinical symptoms and inhibit bone destruction in patients with RA. As early as 1998, the China Food and Drug Administration has approved the TGP for the treatment of RA.^[19]^ TGP have anti-inflammatory, immunomodulatory and analgesic effects. It has been found that the treatment of TGP can reduce the production of pro-inflammatory cytokines (including serum interleukin [IL]-21, tumor necrosis factor [TNF]-α, and IL-6), and also reduce the phosphorylation of nuclear factor (NF)-κ and STAT3 to improve joint destruction in collagen-induced arthritis (CIA) mice.^[20]^ SIN is a prescription drug approved by China Food and Drug Administration for the treatment of RA. Experimental data show that SIN can prevent IL-1β-induced inflammation of human fibroblast-like synovial cells (RAFLS) by inhibiting TLR4/MyD88/NF-κB signal pathway,^[21]^ which indicates that SIN may be a potential drug for the treatment of RA bone injury. GTW and its extract is another effective antirheumatic drug for RA patients who are difficult to be treated by traditional therapy.^[22]^ At present, there is sufficient evidence that GTW extract has immunosuppression, cartilage protective and anti-inflammatory effects on RA.^[23]^ GTW can regulate a variety of cytokines and innate immune cells, including macrophages, dendritic cells, and NK cells, which means that GTW can play an immune-regulation role in the progression of RA bone injury.^[24]^ The level of serum MMP-3 is closely related to the inflammatory response in patients with RA, which are proved to be related to NF- κB signal pathway, TNF-α and IL-6.

Therefore, the combination of MMP-3 level and disease activity in patients with RA can be used as the main reference index to judge the diagnosis and prognosis of bone injury in patients with RA. In recent years, a large number of clinical studies or animal experiment have shown that the effective components of traditional Chinese medicine components combined with MTX can effectively interfere with bone injury in patients with RA.^[25,26]^ However, there is still a lack of evidence-based medicine to prove whether the effective components of traditional Chinese medicine components combined with MTX have a positive effect on bone and joint protection in patients with RA. In summary, we drafted this scheme to analyze and summarize the efficacy and safety of effective components of traditional Chinese medicine combined with MTX in the treatment of bone injury in patients with RA. This scheme will conduct meta-analysis for the existing clinical literature, and provide clear evidence-based medicine in the treatment of bone injury in patients with RA, it can better for clinical treatment.

## Author contributions

**Conceptualization:** Yue Sun, Yucheng Huang.

**Data curation:** Yue Sun, Yucheng Huang, Kexin Lin.

**Formal analysis:** Jiayi Chen, Xueping Li, Yue Sun.

**Funding acquisition:** Yongxiang Gao, Lisha He.

**Methodology:** Lisha He, Zhuozhi Wang.

**Project administration:** Yongxiang Gao, Lisha He.

**Resources:** Yue Sun, Tiantian Chen.

**Software:** Yue Sun, Tiantian Chen, Yucheng Huang.

**Supervision:** Yongxiang Gao.

**Writing – original draft:** Yue Sun, Zhuozhi Wang.

**Writing – review & editing:** Lisha He.
